# Elevated serum interleukin-38 levels in polymyositis and dermatomyositis: diagnostic implications and correlations with inflammatory markers

**DOI:** 10.3389/fimmu.2026.1716099

**Published:** 2026-02-03

**Authors:** Wen Qin, Zhi Li, Guohua Fu, Yan Li, Wenyan Huang, Mengxi Yu, Xiudi Wu, Mingcai Li

**Affiliations:** 1 Department of Rheumatology and Immunology, The First Affiliated Hospital of Ningbo University, Ningbo, China; 2 Department of Pathogenic Biology and Immunology, School of Basic Medical Sciences, Health Science Center, Ningbo University, Ningbo, China; 3 Department of Cardiology, The First Affiliated Hospital of Ningbo University, Ningbo, China

**Keywords:** dermatomyositis, interleukin-38, interstitial lung disease, lactate dehydrogenase, myopathy, polymyositis

## Abstract

**Background:**

Interleukin (IL)-38 has been recently identified as an anti-inflammatory cytokine. Polymyositis (PM) and dermatomyositis (DM) are two prevalent clinical inflammatory myopathies. This study aims to investigate the serum concentrations of IL-38 in patients with PM/DM and their association with these conditions.

**Methods:**

Serum IL-38 levels were quantified using the enzyme-linked immunosorbent assay method in a cohort of 117 subjects, comprising 77 patients with myositis (8 with PM and 69 with DM) and 40 healthy controls. A comprehensive assessment of preliminary clinical characteristics was conducted for each participant through physical examination and review of medical records. The diagnostic utility of IL-38 in PM/DM was evaluated using the receiver operating characteristic curve.

**Results:**

The findings revealed that serum IL-38 concentrations were significantly elevated in patients with PM/DM compared to the control group, irrespective of the presence of interstitial lung disease. No significant difference was observed between the PM and DM groups. Furthermore, IL-38 demonstrated a positive correlation with the visual analogue scale, lactate dehydrogenase (LDH), and other inflammatory markers. Finally, IL-38, either independently or in conjunction with LDH, exhibits significant diagnostic potential.

**Conclusion:**

The study’s findings reveal that patients with PM/DM who present elevated serum levels of IL-38 also show a positive correlation with LDH levels. This suggests IL-38 may participate in modulating inflammatory immune responses in PM/DM and could serve as a potential candidate indicator for these conditions.

## 1 Introduction

Dermatomyositis (DM) and polymyositis (PM) are classified as inflammatory myopathies, characterized by progressive proximal muscle weakness and myalgia, with a gradual onset (1). These conditions can also affect various organs, including the lungs, skin, and heart (2). In DM, dermatological manifestations are particularly pronounced, with symptoms such as photosensitive rashes and Gottron’s papules (2). Interstitial lung disease (ILD) is a major pulmonary complication and a significant contributor to mortality, thereby critically influencing the prognosis of patients with PM/DM (3). Although the precise mechanisms underlying inflammatory myositis remain elusive and its progression is complex, accumulating evidence suggests that the disruption of the balance between pro-inflammatory and anti-inflammatory cytokines plays a crucial role in the muscle and organ pathology associated with myositis (4–6).

The nuclear factor (NF)-κB serves as a central regulator of immune and inflammatory responses. Its translocation to the nucleus and subsequent expression of target genes facilitate ubiquitination and proteasome-mediated protein degradation, thereby modulating the effects of inflammatory cytokines, particularly tumor necrosis factor (TNF)-α and interleukin (IL)-6, on muscle atrophy (7). Patients with myositis exhibit elevated levels of IL-1α and IL-1β in their endothelial cells, which leads to increased expression of IL-6 and adhesion molecules, as well as the promotion of leukocyte migration, further exacerbating the localized immune response (8, 9). The IL-1 receptor (IL-1R) is widely distributed in muscle fibers, endothelial cells, and inflammatory cells, with its expression levels showing an upward trend in patients with myositis (10). Furthermore, Chabaud et al. (11) have corroborated the synergistic effect of IL-1, TNF-α, and IL-17 on IL-6 production. Moreover, IL-17 facilitates the production of IL-1 and TNF-α, thereby contributing to the disease’s pathogenic process. Other members of the IL-1 family (IL-1F) also play a critical role in the onset and progression of myositis. For instance, IL-18 can synergize with IL-2 to enhance IFN-γ expression. Simultaneously, elevated levels of IL-18 may result in immune regulatory dysfunction (12). Recently, Jiang et al. (13) reported significantly increased serum IL-35 concentrations in patients diagnosed with PM/DM compared to healthy controls. Subsequent experiments demonstrated that the secretion of IL-17 and TNF-α by PBMCs from PM/DM patients, in response to LPS stimulation, could be markedly inhibited by the addition of IL-35. These findings suggest that IL-35 may exert an anti-inflammatory effect in PM/DM. In the pathological mechanisms of myositis, cytokines such as IL-23 (14), IL-15 (15), and IL-37 (16) are also pivotal.

IL-38, a recent addition to the IL-1F, plays a role in regulating autoimmune disorders and provides protection against inflammatory responses (17). IL-38 is synthesized by epithelial cells, monocytes, macrophages, and various other immune cells (7, 18, 19), and is found in tissues such as the spleen, skin, and thymus (20, 21). Initially identified and named by Lin et al. in 2001 through high-throughput cDNA screening, IL-38 was originally termed IL-1HY2 (22). The gene encodes a precursor protein consisting of 152 amino acids, with a molecular weight of 17 kDa. Notably, the amino acid sequence of IL-38 shares 41% identity with IL-1 receptor antagonist (IL-1Ra) and 43% identity with IL-36Ra (22, 23). Furthermore, IL-38 has the ability to interact with specific receptors, including IL-36R, IL-1R1, and IL-1 receptor accessory protein-like 1 (IL-1RAPL1), to execute its biological functions (21, 24). Similar to other members of the IL-1F family, IL-38 becomes biologically active following protease treatment of its N-terminal (25). Nonetheless, the involvement of full-length and truncated recombinant IL-38 proteins in activating the IL-1R1 pathway remains a subject of debate (26, 27). These recombinant IL-38 proteins are capable of binding to IL-1RAPL1, thereby modulating inflammatory processes (26). Ongoing research has demonstrated that IL-38 holds significant potential in the treatment of autoimmune and inflammatory diseases, including gout (28), psoriasis (29), systemic lupus erythematosus (30), and rheumatoid arthritis (31). Concurrently, IL-38 is capable of interacting with inhibitory co-receptors and inhibiting the downstream NF-κB and MAPK signaling pathways, thereby suppressing inflammatory responses and exhibiting its anti-inflammatory properties (32).

In summary, IL-38 is likely to play a crucial role in the pathological progression of PM and DM through its interaction with specific receptors. It also has the potential to serve as an important candidate indicator for myositis patients, providing essential support for clinical diagnosis, treatment decisions, and prognostic assessments.

## 2 Materials and methods

### 2.1 Participants and study design

**Figure 1:**
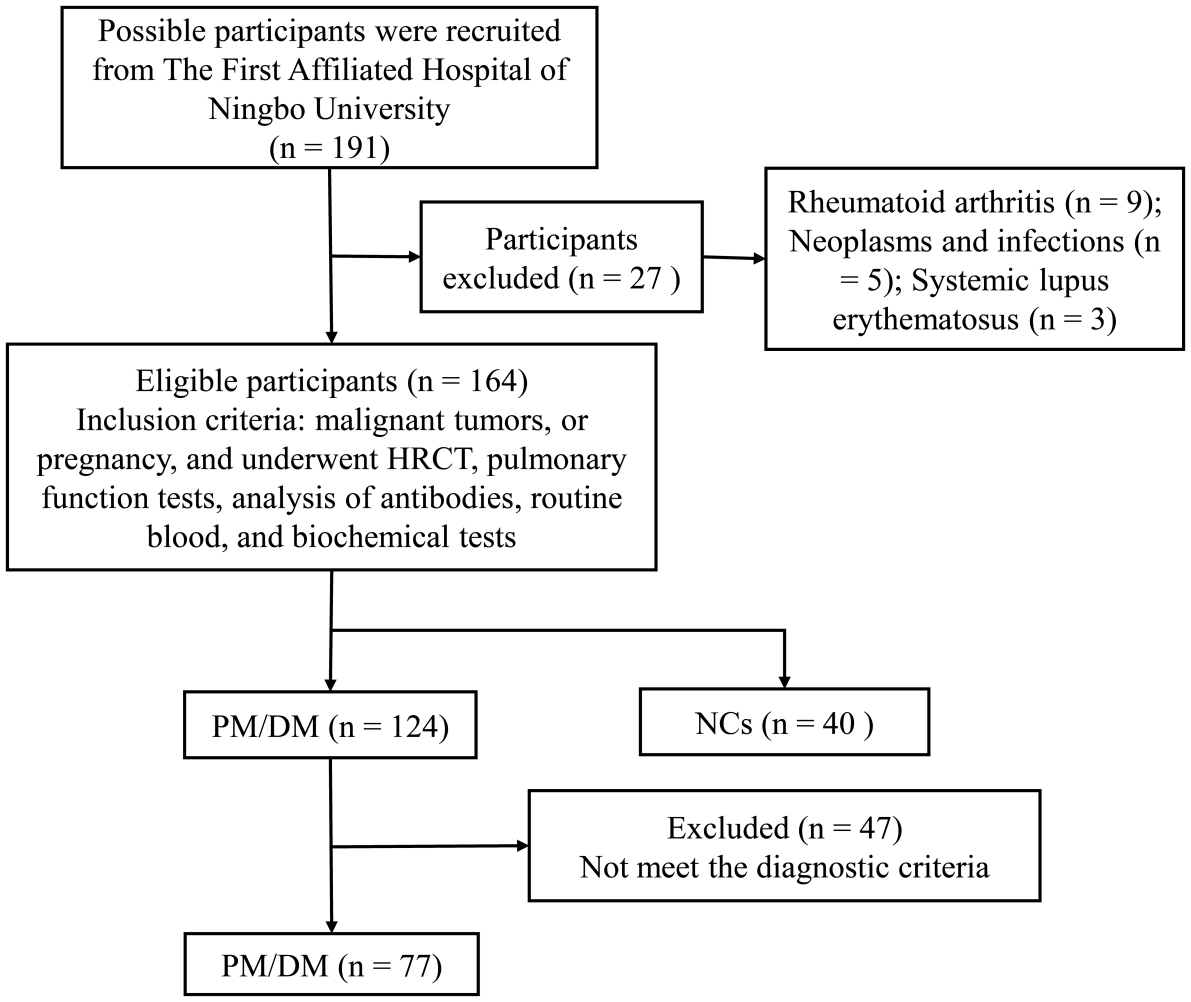
Participants recruitment process. PM, polymyositis; DM, dermatomyositis; NCs, negative controls; HRCT, high-resolution computed tomography.

From the cohort of patients admitted to the First Affiliated Hospital of Ningbo University between September 2023 and October 2024, 77 myositis patients (comprising 8 with PM and 68 with DM, and 1 immune mediated necrotizing myopathy) over the age of 18 were enrolled based on the diagnostic criteria established by Bohan and Peter (1, 33) and were matched with 40 negative controls (NCs). Disease duration was defined as the interval from the date of the first myositis diagnosis to the date of serum collection; these data are presented in **Table 1**. Patients diagnosed with PM or DM were further categorized into two distinct groups: an ILD group (n = 54) and a non-ILD group (n = 23). The detailed screening process for subjects is illustrated in **Figure 1**.

**Table 1:** Characteristics of subjects.

Variables	PM/DM		NCs	*P*-value
	Without ILD	With ILD		
N	23	54	40	/
Disease duration(months)	25.00 (12.00, 37.00)	35.00 (13.00, 52.50)	/	/
Demographic data				
Female, n (%)	18 (78.26)	43 (79.63)	30 (75.00)	0.6431
Age (y)	54.00 (39.00, 62.00)	55.00 (50.00, 62.00)	51.50 (42.25, 62.75)	0.3767
PM, n (%)	6 (26.09)	2 (3.70)	/	/
DM, n (%)	17 (73.91)	52 (96.29)	/	/
Laboratory indicators				
VAS score	3.00 (2.00, 6.00)	4.00 (3.00, 5.00)	/	0.7503
CRP (mg/L)	0.50 (0.30, 1.90)	1.53 (0.44, 15.20)	/	0.0086
ESR (mm/h)	12.00 (6.00, 23.00)	25.00 (16.00, 44.50)	/	0.0005
IgG (g/L)	11.20 (9.09, 13.80)	14.70 (11.65, 17.23)	/	0.0011
CK (U/L)	86.00 (55.00, 260.00)	89.50 (69.75, 166.25)	85.00 (67.25, 116.75)	0.4219
LDH (U/L)	250.00 (173.00, 328.00) ^*^	244.00 (207.75, 276.25) ^#^	163.15 (130.00, 182.00)	<0.0001
TBIL (μmol/L)	9.10 (7.80, 12.40)	8.45 (7.08, 11.98)	11.30 (8.50, 14.03)	0.0344
ALB (g/L)	41.90 (40.10, 43.80)	40.80 (34.70, 42.95) ^#^	43.80 (42.43, 45.58)	<0.0001
WBC (10^9^/L)	6.40 (5.60, 7.90)	7.90(6.00, 10.20) ^#^	5.40 (4.80, 6.70)	<0.0001
EOP(%)	62.40 (57.80, 69.10)	67.40 (58.60, 77.33) ^#^	56.40 (48.78, 61.83)	0.0018
BAP (%)	0.40 (0.30, 0.50)	0.40 (0.20, 0.60) ^#^	0.60 (0.40, 0.68)	0.0039
LY (10^9^/L)	1.54 ± 0.62	1.71 ± 0.81	1.86 ± 0.46	0.0342
RBC (10^9^/L)	4.46 ± 0.49	4.37 ± 0.58 ^#^	4.59 ± 0.38	0.0176
ANA	14 (60.87)	44 (81.48)	/	/
MSAs (myositis-specific autoantibodies), n (%)				
Anti-Jo-1	2 (8.70)	13 (24.07)	/	/
Anti-OJ	1 (4.35)	2 (3.70)	/	/
Anti-PL-7	0 (0.00)	5 (9.26)	/	/
Anti-TIF-1γ	5 (21.74)	1 (1.85)	/	/
Anti-MDA-5	1 (4.35)	11 (20.37)	/	/
Anti-Mi-2	3 (13.04)	4 (7.41)	/	/
Anti-NXP2	2 (8.70)	1 (1.85)	/	/
Anti-SRP	0	2 (3.70)	/	/
Anti-PL-12	0	3 (5.56)	/	/
Anti-PL-17	0	2 (3.70)	/	/
Anti-EJ	0	2 (3.70)	/	/
Anti-SAE1	0	1 (1.85)	/	/
MAAs (myositis-associated autoantibodies), n (%)				
Anti-Ro-52	4 (17.39)	29 (53.70)	/	/
Anti-PM-Scl	1 (4.35)	2 (3.70)	/	/
Anti-Ku	2 (8.70)	3 (5.56)	/	/
Anti-U1-nRNP	1 (4.35)	0	/	/
Autoantibodies-negative	6 (26.10)	4 (7.41)	/	/
Treatment, n (%)				
Corticosteroid	21 (91.30)	52 (96.29)	/	/
Immunosuppressant	18 (78.26)	40 (74.07)	/	/
Others	4 (17.39)	14 (25.93)	/	/
No therapy	1 (4.35)	0 (0.00)	/	/

PM, polymyositis; DM, dermatomyositis; NCs, negative controls; ILD, interstitial lung disease; VAS, visual analogue scale; CRP, C-reactive protein; ESR, erythrocyte sedimentation rate; IgG, immunoglobulin G; CK, creatine kinase; LDH, lactate dehydrogenase; TBIL, total bilirubin; ALB, albumin; WBC, white blood cell count; EOP, eosinophil percentage; BAP, basophil percentage; LY, lymphocyte count; RBC, red blood cell count; ANA, antinuclear antibody; MDA-5, melanoma differentiation-associated gene 5. The *P*-value was obtained by one-way analysis of variance, Kruskal–Wallis test, Mann–Whitney *U* test. ^*^PM/DM patients without ILD group compared with HC, *P* < 0.05. ^#^PM/DM patients with ILD group compared with HC, *P* < 0.05.

NCs were recruited from outpatients. The inclusion and exclusion criteria were as follows (1): No apparent signs of illness during routine screenings (2); Age 18 or older (3); History of hormone use, susceptibility to infections, presence of tumors, autoimmune disorders, and other illnesses, with particular emphasis on myositis.

All participants provided informed consent, and the study received approval from the Medical Ethics Committee of The First Affiliated Hospital of Ningbo University.

### 2.2 Clinical indicators

Data on age and sex for each study participant were collected prior to their inclusion. Statistical analysis indicated no significant differences in age and sex compositions between PM/DM patients and the NCs (P = 0.3767, P = 0.6431). The visual analogue scale (VAS) was employed to assess the disease activity of myositis, which was physician-derived: experienced rheumatologists in our research team, familiar with the clinical manifestations and activity assessment standards of PM and DM, conducted comprehensive evaluations of each patient’s clinical symptoms (such as muscle weakness, myalgia, skin lesions, and systemic involvement) and assigned VAS scores based on unified and standardized assessment criteria. Additional clinical characteristics of individuals diagnosed with PM/DM were also documented. ILD was assessed using high-resolution computed tomography (HRCT). Blood tests, including C-reactive protein (CRP), erythrocyte sedimentation rate (ESR), immunoglobulin G (IgG), lactate dehydrogenase (LDH), were conducted using automatic hematology analyzer (XN-2000, SYSMEX) and biochemical analyzer (AU2700, OLYMPUS OPTICAL Co.). Antinuclear antibody (ANA) was detected by indirect immunofluorescence on HEp-2 cells using the EUROIMMUN IIFT kit (EUROIMMUN (Hangzhou) Medical Laboratory Diagnostics Co., Ltd.; positive at ≥ 1:80), and myositis-specific/-associated antibodies were tested by EUROIMMUN line-blot from the same manufacturer (bands ≥ 1 + considered positive).

### 2.3 Quantification of serum IL-38

The serum concentration of IL-38 was quantified using an enzyme-linked immunosorbent assay (ELISA) kit (CSB-EL011615HU, Cusabio Technology, Wuhan, China), following the protocol provided by the manufacturer. The ELISA kits exhibited inter-assay and intra-assay coefficients of variation of less than 8% and 10%, respectively. The sensitivity threshold of the assay was set at a range of 31.25 to 2,000 pg/mL.

The experimental procedure was as follows: thawed serum samples and diluted standards were added to an enzyme-coated plate, pre-equilibrated to room temperature, and incubated for 30 min. After a 2-hour incubation at 37°C, the supernatant was removed. Subsequently, a biotinylated antibody and horseradish peroxidase-conjugated avidin were added and incubated for an additional 1 h at 37°C. The reaction was then developed with 3, 3’, 5, 5’-tetramethylbenzidine (TMB) for 30 min in the dark and terminated by adding a stop solution. Absorbance was measured at 450 nm using a spectrophotometer (Multiskan GO; Thermo Fisher Scientific). A standard curve was generated using serially diluted standard solutions.

### 2.4 Statistical analysis

The data analysis was conducted using IBM SPSS Statistics version 24.0. Graphical representations of the analysis results were produced using GraphPad Prism version 9.0, which also facilitated the execution of statistical analyses for this study. The quantitative data were subjected to normality testing, with normally distributed data presented as mean ± standard deviation, whereas the median (interquartile range) was applied to represent the non-normal data. Categorical data were expressed as frequencies. For the appraisal of the three data sets, a one-way analysis of variance (ANOVA) or the Kruskal–Wallis test was implemented. The Mann–Whitney U test was utilized for the comparison of quantitative values in the case of unpaired data, whereas the Student’s t-test was applied for paired data. Analyses of the clinical data correlations were conducted employing rank correlations. The diagnostic significance of the disease was shown through the area under the curve (AUC) of the receiver-operating characteristic (ROC) curves. All tests were two-tailed, with a significance level of 0.05.

## 3 Results

### 3.1 Clinical characteristics of subjects

The demographic characteristics, including gender and age, were comparable across the three groups (*P* = 0.3767, *P* = 0.6431). There were no significant differences in serum CK levels (*P* = 0.4219) and VAS scores (*P* = 0.7503) among the study participants. Pairwise comparisons using the Kruskal–Wallis test indicated that all other indicators for patients with PM/DM differed significantly from those of NCs, with the exceptions of TBIL and LY. However, only LDH levels were significantly different in the PM/DM group without ILD. The analysis of autoantibody profiles showed that anti-Ro-52 and ANA were the most prevalent among patients with myositis. Of the 80 patients, 79 received therapeutic interventions, while one patient did not undergo any treatment. Detailed clinical data are provided in **Table 1**. Glucocorticoids comprised prednisone and methylprednisolone, immunosuppressants included methotrexate, mycophenolate mofetil, tofacitinib, cyclophosphamide, etc., and adjunctive agents such as total glucosides of paeony and hydroxychloroquine.

### 3.2 Serum IL-38 levels

**Figure 2:**
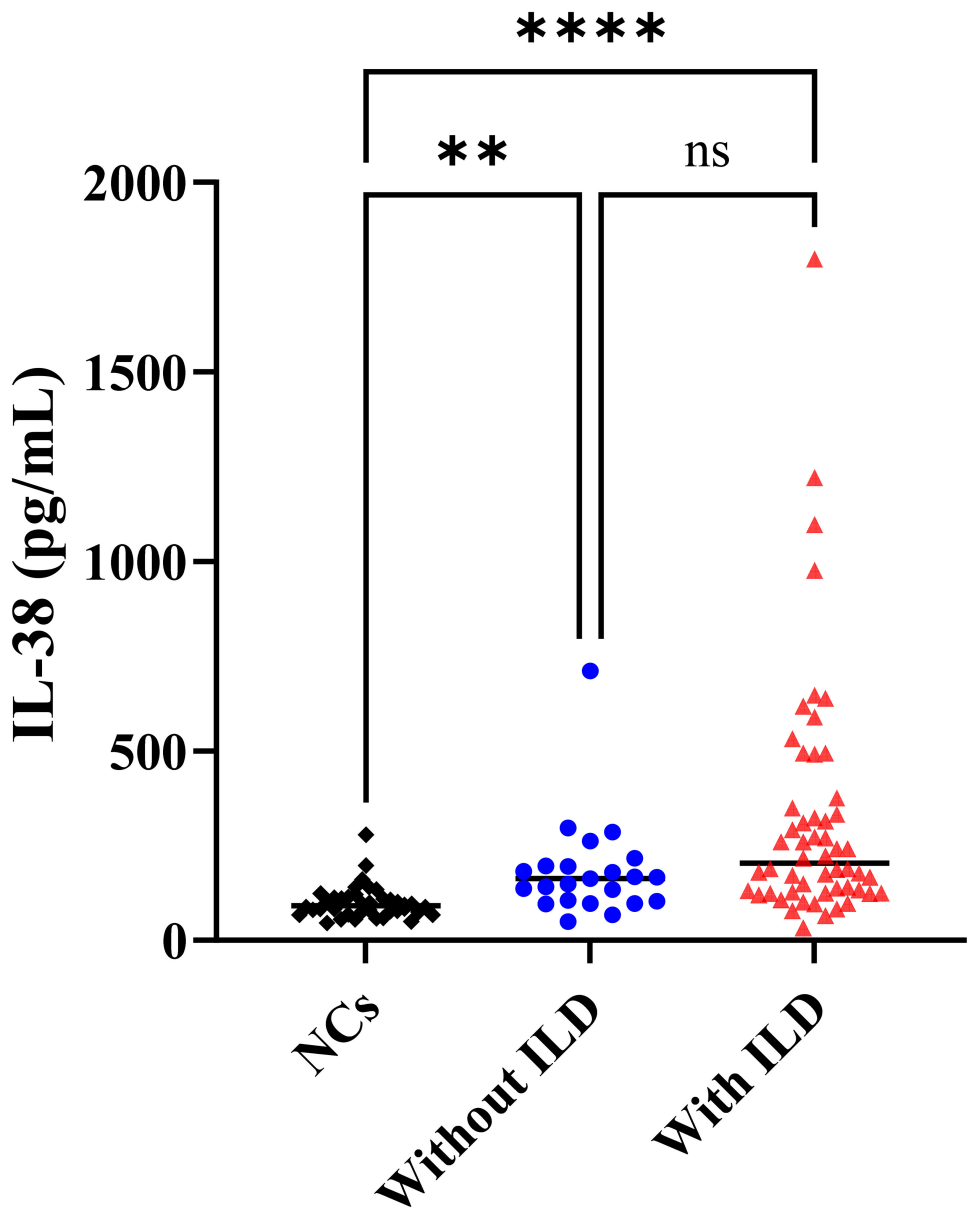
Serum IL-38 concentration in NCs, PM/DM patients without ILD, and PM/DM patients with ILD. NCs, negative controls; PM, polymyositis; DM, dermatomyositis; ILD, interstitial lung disease. The *P*-value was derived from the Kruskal–Wallis test. Standard deviations for each group are indicated as “mean ± SD” as follows: NCs: 99.77 ± 43.00 pg/mL; PM/DM without ILD: 182.75 ± 132.30 pg/mL; PM/DM with ILD: 322.35 ± 324.83 pg/mL. **P < 0.01, ****P < 0.0001, ns, no significance.

The serum IL-38 levels of the subjects are illustrated in **Figure 2**. The IL-38 concentrations in PM/DM patients without ILD, PM/DM patients with ILD, and NCs were 162.85 (103.26, 195.92), 203.61 (126.72, 356.05), and 91.06 (75.35, 113.91) pg/mL, respectively. The Kruskal–Wallis test identified significant variations in IL-38 levels among the three groups. Subsequent Dunn’s *post-hoc* analysis revealed that patients with PM/DM and ILD exhibited significantly elevated concentrations of IL-38 compared to both NCs (*P* < 0.0001) and PM/DM patients without ILD (*P* = 0.0014). However, no significant difference was observed between the latter two groups (*P* = 0.3018).

Furthermore, IL-38 concentrations were significantly higher in PM/DM patients with elevated LDH (LDH > 250 U/L) compared to those with normal LDH levels (*P* = 0.0043). In contrast, no statistically significant differences in IL-38 concentrations were observed between patients with or without anti-Ro-52 antibodies (*P* = 0.1540) or ANA (*P* = 0.3802). **Table 2** provides a detailed comparative analysis of serum IL-38 concentrations across these subgroups, based on LDH levels, anti-Ro-52 antibody status, and ANA presence.

**Table 2:** Association between IL-38 levels and LDH as well as autoantibodies in patients with PM/DM.

Variables	n	IL-38 (pg/mL)	*P*-value
LDH			0.0389
normal	41	166.34 (121.71, 240.97)	
Increased	36	241.60 (138.06, 493.01)	
Anti-Ro-52			0.1540
Positive	33	195.92 (148.80, 312.13)	
Negative	44	157.54 (105.58, 295.84)	
ANA			0.3802
Positive	58	187.92 (124.90, 325.56)	
Negative	19	168.09 (125.36, 217.25)	

LDH, lactate dehydrogenase; ANA, antinuclear antibody. The *P*-value was determined through the application of Mann–Whitney *U* test.

### 3.3 Correlation analysis

**Figure 3:**
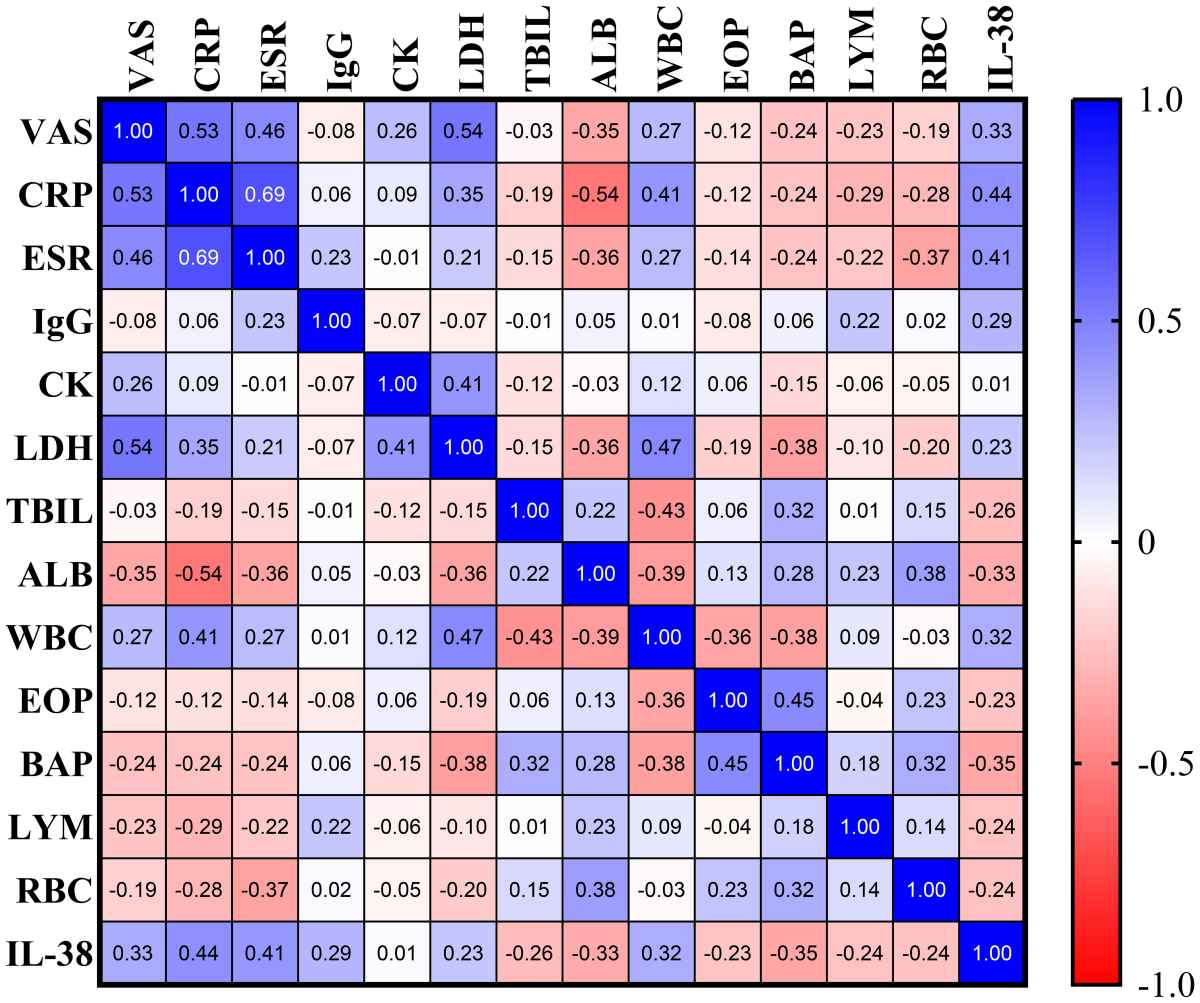
Correlation heat map of all indicators in PM/DM patients. VAS, visual analogue scale; CRP, C-reactive protein; ESR, erythrocyte sedimentation rate; IgG, immunoglobulin G; CK, creatine kinase; LDH, lactate dehydrogenase; TBIL, total bilirubin; ALB, albumin; WBC, white blood cell count; EOP, eosinophil percentage; BAP, basophil percentage; LY, lymphocyte count; RBC, red blood cell count.

**Figure 4:**
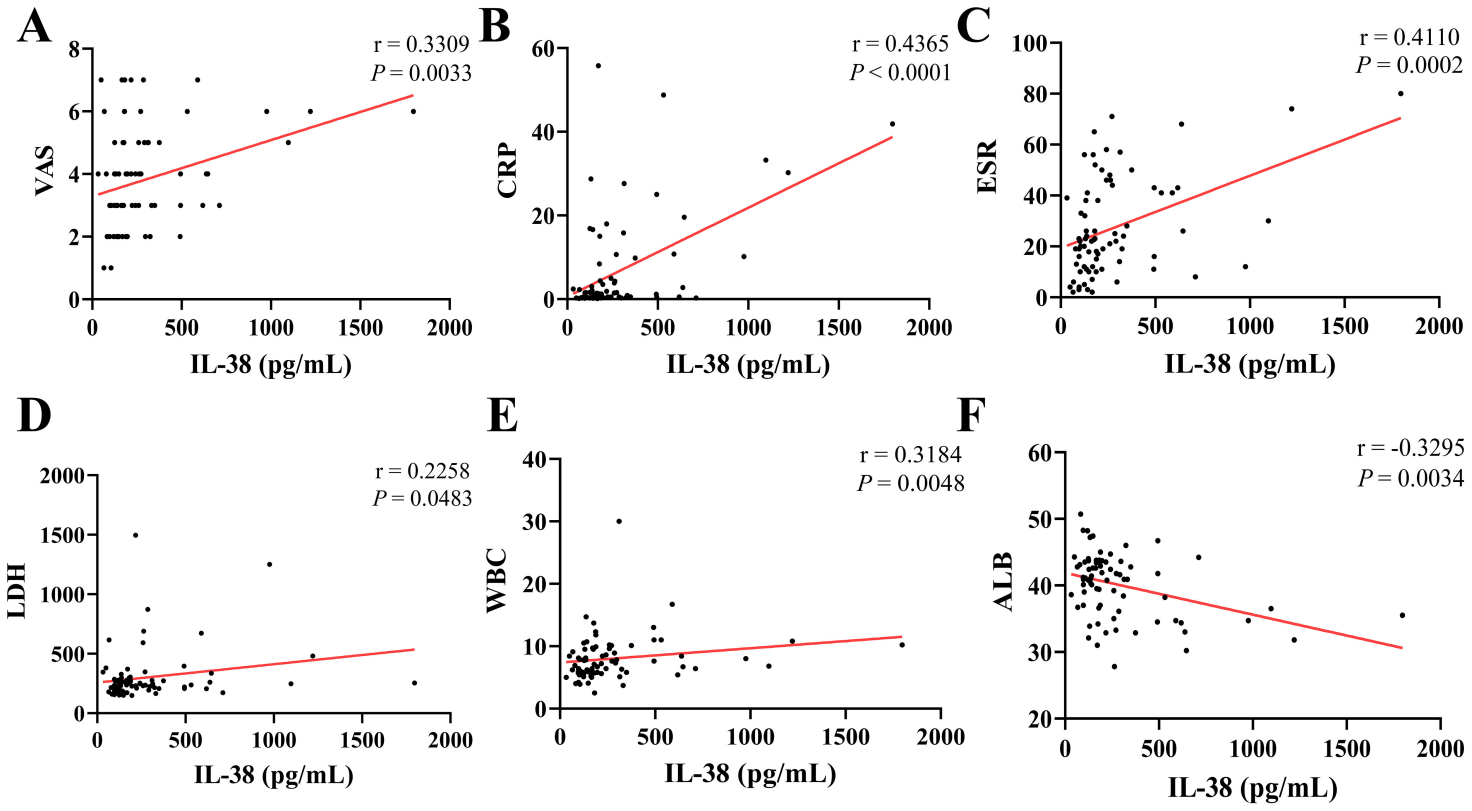
Correlation analysis of various laboratory indicators.Correlations of IL-38 levels with VAS **(A)**, CRP **(B)**, ESR **(C)**, LDH **(D)**, WBC **(E)**, and ALB **(F)**. VAS, visual analogue scale; CRP, C-reactive protein; ESR, erythrocyte sedimentation rate; LDH, lactate dehydrogenase; WBC, white blood cell count; ALB, albumin.

The Spearman correlation method was employed to examine the relationships between various laboratory parameters, as depicted in **Figure 3**. In PM/DM patients, IL-38 levels demonstrated a significant positive correlation with VAS scores (r = 0.3309, *P* = 0.0033) (**Figure 4A**), CRP levels (r = 0.4365, *P* < 0.0001) (**Figure 4B**), ESR (r = 0.4228, *P* = 0.0002) (**Figure 4C**), LDH (r = 0.2258, *P* = 0.0483) (**Figure 4D**), WBC (r = 0.3184, *P* = 0.0048) (**Figure 4E**), and a significant negative correlation with ALB (r = -0.3295, *P* = 0.0034) (**Figure 4F**).

### 3.4 ROC curve analysis

**Figure 5:**
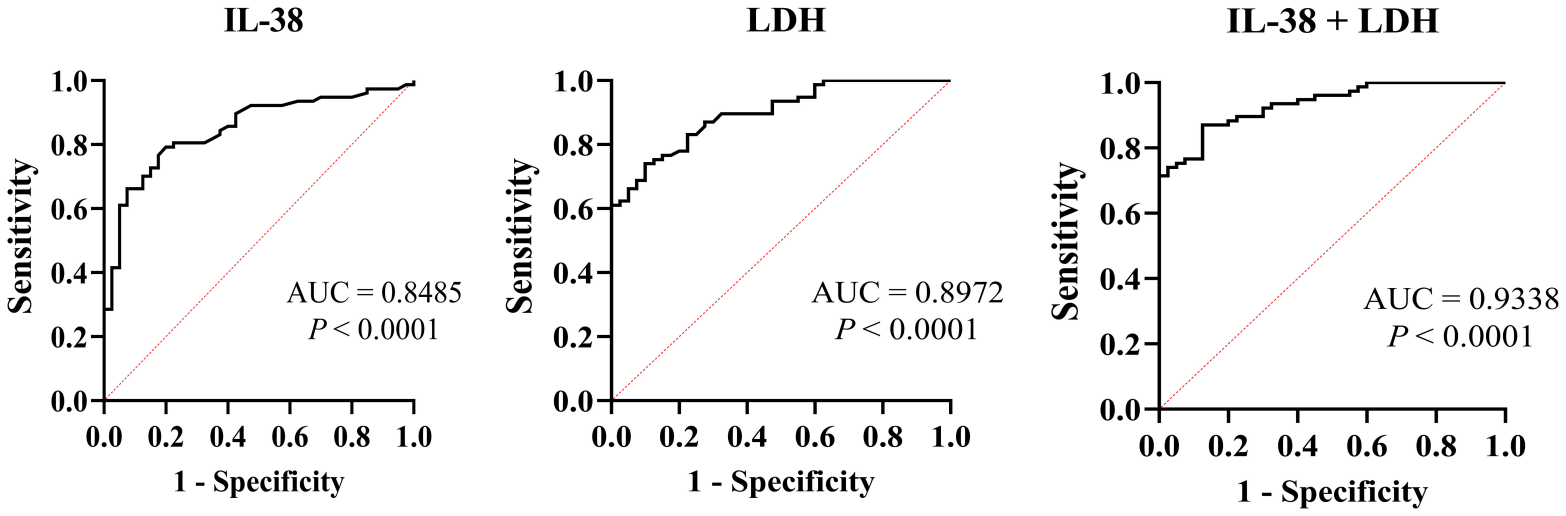
The ROC curves of IL-38, LDH, and IL-38 combined with LDH were used to diagnose PM/DM. ROC, receiver operating characteristic; AUC, area under the curve; LHD, lactate dehydrogenase.

The ROC curve analysis, used to evaluate the diagnostic value of IL-38 and LDH levels in PM/DM, showed AUC values of 0.8454 and 0.8972, respectively. The combined assessment of these two biomarkers resulted in an increased AUC of 0.9338, indicating enhanced diagnostic accuracy (**Figure 5**).

### 3.5 Multivariate logistic regression for PM/DM

**Table 3** presents the logistic regression models for participant variables, highlighting those with P-values below 0.05. **Table 4** illustrates the outcomes of the multinomial logistic regression analyses, confirming LDH and IL-38 as independent risk predictors for PM/DM.

**Table 3:** Single factor analysis of participants.

Variables	PM/DM (n = 77)	NCs (n = 40)	*P*-value
LDH (U/L)	247.00 (199.00, 287.00)	163.15 (130.00, 182.00)	<0.0001
TBIL (μmol/L)	8.90 (7.25, 12.35)	11.30 (8.50, 14.03)	0.0344
ALB (g/L)	40.13 ± 4.80	43.94 ± 2.18	<0.0001
WBC (10^9^/L)	7.60 (5.80, 9.65)	5.40 (4.80, 6.70)	<0.0001
EOP (%)	64.70 (58.30, 76.40)	56.40 (48.78, 61.83)	0.0018
BAP (%)	0.40 (0.20, 0.60)	0.60 (0.40, 0.68)	0.0039
LY (10^9^/L)	1.66 ± 0.76	1.86 ± 0.46	0.0342
RBC (10^9^/L)	4.40 ± 0.56	4.59 ± 0.38	0.0176
IL-38 (pg/mL)	180.21 (125.36, 303.72)	91.06 (75.35, 113.91)	<0.0001

NCs, negative controls; PM, polymyositis; DM, dermatomyositis; LDH, lactate dehydrogenase; TBIL, total bilirubin; ALB, albumin; WBC, white blood cell count; EOP, eosimophil percentage; BAP, basophil percentage; LY, lymphocyte count; RBC, red blood cell count; The *P*-value was obtained by Mann–Whitney *U* test.

**Table 4:** Multivariate logistics regression analysis of laboratory indicators in PM/DM patients.

Variables	Odds Ratio (95% CI)	*P*-value
LDH (U/L)	1.040 (1.019, 1.062)	0.0002
ALB (g/L)	0.793 (0.638, 0.987)	0.0374
IL-38 (pg/mL)	1.019 (1.004, 1.038)	0.0110

LDH, lactate dehydrogenase; ALB, albumin; The *P*-value was calculated through logistic regression.

## 4 Discussion

Although the precise etiology of PM/DM remains elusive, cytokines play a crucial role in the disease’s progression, characterized by a dynamic interaction between pro-inflammatory and anti-inflammatory cytokines. IL-38, known for its anti-inflammatory properties (34), functions as a cytokine with regulatory effects on various immune diseases (35, 36), though its specific role in PM/DM is yet to be elucidated. Research findings indicate that patients with PM/DM exhibit significantly elevated serum levels of IL-38 compared to the negative control group. ILD is the most common and severe extramuscular manifestation in PM/DM, significantly affecting patient prognosis. Previous studies have identified that PM/DM with ILD constitutes a distinct immunopathological endotype, characterized by marked activation of the type I interferon pathway (37–39). The differential expression of IL-38, a known negative regulator of the immune response, in this specific context remains unclear. To address this, we analyzed the 70% of our cohort diagnosed with ILD, comparing their serum IL-38 levels and inflammatory indices to those of patients without pulmonary involvement. This methodological approach minimized ILD-related confounding factors, enabling a more precise evaluation of the diagnostic value of IL-38 and its complex relationship with systemic inflammation in PM/DM. Furthermore, elevated IL-38 levels were observed in individuals with increased LDH levels compared to those with LDH levels within the normal range. LDH serves as an indicator of muscle damage. The observed positive correlation between serum IL-38 and LDH suggests that IL-38 levels increase concomitantly with heightened disease activity in inflammatory myopathies. We propose that this pattern signifies the activation of an intrinsic negative-feedback mechanism: as muscle damage and inflammation escalate, the anti-inflammatory mediator IL-38 is released in a compensatory fashion. Consequently, elevated serum IL-38 may be interpreted as a biomarker reflecting the immune system’s attempt to reestablish homeostasis during active disease, rather than as a direct agent of tissue damage. The precise cellular origins, regulatory pathways, and local functions of IL-38 within the affected muscle microenvironment warrant further investigation through future histological studies. Correlation analyses revealed a positive association between serum IL-38 levels and markers of disease activity, such as ESR, CRP, and VAS. Additionally, a positive correlation was identified with the skeletal muscle enzyme LDH and the non-specific inflammatory marker WBC, while a negative correlation was noted with ALB. ROC curve analysis demonstrated an AUC value of 0.8485 for IL-38, underscoring its diagnostic significance for PM/DM. The combined sensitivity of IL-38 and LDH was 0.9338, surpassing the sensitivity of LDH alone. Logistic regression analysis revealed a significant association between elevated serum IL-38 concentrations and disease activity in patients with PM/DM.

These findings suggest that IL-38 plays a crucial role in the inflammatory response associated with PM/DM, aiding in the assessment of disease severity or muscle damage in affected individuals. Elevated levels of IL-38 can be induced by specific activators. In a mouse model of collagen-induced arthritis, IL-38 significantly reduces the clinical severity of inflammation (40), as well as decreases macrophage infiltration and the expression of IL-17, IL-22, IL-23, and TNF-α. IL-37, another member of the IL-1F, also functions as an anti-inflammatory cytokine. Its expression can be induced by TNF-α through the activation of NF-κB (41), and it can be further upregulated by cytokines such as IL-1β and IL-10 (41). In an experimental myositis mouse model, IL-37 significantly mitigated the histological damage to muscle and lung tissues caused by PTX stimulation (16), and it also reduced the levels of several pro-inflammatory cytokines, including IL-1β, IL-6, and TNF-α. Consequently, IL-38 demonstrates anti-inflammatory properties similar to those of IL-37, suggesting its potential role in a cytokine-mediated positive feedback mechanism that enhances IL-38 levels. In a study involving patients with systemic lupus erythematosus (SLE), it was observed that the serum concentration of IL-38 was significantly elevated in SLE patients compared to healthy individuals (26). Further analysis demonstrated that individuals with active disease exhibited higher concentrations of IL-38 expression compared to those with inactive disease Further analysis revealed that individuals with active disease exhibited higher concentrations of IL-38 expression than individuals with inactive disease (26). Consequently, we hypothesize that elevated IL-38 may function as an anti-inflammatory cytokine, initiating a protective anti-inflammatory response in PM/DM. This response could potentially mitigate tissue and organ damage by counteracting deleterious factors. However, the precise mechanisms underlying this process remain incompletely understood.

This study represents a pioneering effort to investigate the differences in IL-38 concentrations between myositis patients and negative controls. Additionally, we stratified PM/DM patients into two groups, with and without ILD, to examine variations in serum IL-38 concentration under these distinct conditions. Correlation analysis and ROC curve analysis were employed to evaluate the potential role of IL-38 in the pathogenesis of PM/DM and its diagnostic significance. It should be noted that this single-center study included a limited number of myositis subgroups, which inherently constrains the generalizability of the findings. Despite utilizing objective indicators rather than subjective recollections, some degree of bias is unavoidable. We do not believe that the presence of bias compromises the reliability of our findings. Secondly, the absence of standardized IMACS core set measures limits the ability to conduct a detailed analysis of the relationship between IL-38 levels and disease activity. Thirdly, this study did not perform immunohistochemical analysis to assess IL-38 expression in muscle tissues from patients with PM or DM. Consequently, we were unable to evaluate its local expression within the affected muscles, highlighting an important avenue for future research. Additionally, although our study was limited by a small sample size, we conducted comprehensive subgroup analyses. Future research should incorporate standardized IMACS evaluations, utilize large multi-center samples, and systematically collect complete clinical data from patients with various myositis subtypes. This approach would facilitate a deeper exploration of the differences and clinical significance of IL-38 in myositis and further validate its potential value.

In conclusion, our study identifies a significant increase in IL-38 concentrations in the serum of individuals diagnosed with PM/DM. While the exact role of IL-38 in PM/DM is not yet fully understood, preliminary evidence suggests that it may play a role in disease progression and modulate associated inflammatory processes. Consequently, IL-38 may have potential clinical diagnostic and therapeutic value for PM/DM.

## Data Availability

The raw data supporting the conclusions of this article will be made available by the authors, without undue reservation.
